# Growth parameters and calcium homeostasis in cystic fibrosis patients with CFTR I1234V mutation

**DOI:** 10.4103/0256-4947.57176

**Published:** 2009

**Authors:** Atqah Abdul Wahab, Ashraf Soliman, Mohamed O. A. Rahman

**Affiliations:** a From the Department of Pediatrics, Hamad Medical Corporation, Doha, Qatar; b From the Department of Biochemistry, Hamad Medical Corporation, Doha, Qatar

**To the Editor:** Cystic fibrosis (CF) is the most common life-threatening recessive genetic trait among whites.^1^ Despite a recent emphasis on adequate nutrition, impaired growth occurs frequently in children with CF and the etiology appears to be multi-factorial.^1^ Pancreatic insufficiency (PI) can be a major factor,^2^ but other factors including insulin resistance, bone disease, hepatobiliary dysfunction and vitamin D deficiency may be significant risk factors for defective bone growth.^3^ The cystic fibrosis transmembrane conductance regulator (CFTR) I1234V mutation, with normal exocrine pancreatic function, is a common mutation among Arabs in the Arabian Gulf region.^4^

Growth and nutritional parameters and FEV_1_ were measured in 40 CF patients with CFTR I1234V mutation, (22 males and 18 females) from 22 families arising from a single large Arab kindred tribe attending the CF clinic at Hamad Medical Corporation, Qatar. The majority of patients were not taking regular nutritional supplements or vitamins. A single venous blood sample was obtained from each patient for measurement of circulating concentrations of 25-hydroxy vitamin D (25OHD) using DiaSorin 25-OHD radioimmunoassay kits (DiaSorin, Inc., Stillwater, Minnesota, USA), calcium, phosphorus, alkaline phosphatase (ALP), parathyroid hormone (PTH) intact molecule assayed by the electrochemiluminescence immunoassay [ECLIA] method (Elecsys 2010, Roche, Germany), and albumin. FEV_1_ was measured with a flow-sensing spirometer (Sensor Medicus Model V6200, Germany). The mean height standard deviation score (HtSDS) was −0.46±1.0 and mean (SD) body mass index (BMI) was 20.8 (5.8) at a mean (SD) age of 11.9 (6.18) years. No patient showed clinical signs of rickets or osteomalacia. All patients had normal stool elastase activity, normal serum calcium with the median of 2.47 mmol/L (range, 2.21-2.66 mmol/L), phosphorus 1.54 mmol/L (range, 0.9-2.19 mmol/L), and ALP 171 U/L (range, 44-382 U/L). The mean (SD) circulating 25OHD concentration was 21.25 (3.42) ng/mL with 60% of patients <20 ng/mL (reference values, ≤10 ng/mL severe deficiency, 10-20 ng/mL mild to moderate deficiency and >20-80 ng/mL optimal). A mild elevation of serum ALP was found in 5/40 and increased serum PTH (67-77 pg/mL; normal range 9-65 pg/mL) was detected in 6 patients. The mean (SD) concentration of 25OHD was lower in adolescents >12 years of age vs those ≤12 years (15.8±7.63 vs 24.067±13.21 ng/mL). Moreover, circulating concentrations of phosphate and ALP were lower in the adolescents (>12 years) versus children ≤12 years (1.31±0.22 vs 1.84±0.23 mmol/L and 132.70±62.56 vs 229.40±62.57 U/L), respectively. Serum PTH concentration was lower in young children (<5 years) vs older children (≥5 years) and adolescents with CF (36.40±22.72, 48.43±14.03 and 47.75±16.85 pg/mL, respectively). HtSDS and FEV_1_ showed no difference between patients with or without vitamin D deficiency (*P*=.76 and 0.2, respectively). HtSDS was correlated with FEV_1_ (r=0.30; *P*<.05) and ALP (r=0.57; *P*<.01). Neither FEV_1_ nor BMI correlated with 25OHD concentrations.

The high prevalence of vitamin D deficiency in our CF patients, despite normal pancreatic exocrine function, might be related to the hot weather and the usual clothing that covers most of the body, decreasing effective sun exposure. An intermittent increase of PTH secretion during vitamin D deficiency (n=6) is known to be an adaptive mechanism to increase alpha-hydroxylation of vitamin D and to maintain both normal serum calcium concentration and bone growth. However, when PTH levels become excessive or persistently high for long periods, osteopenia/osteoporosis may occur.

Our adolescent patients with CF had significantly lower serum levels of calcium, phosphate and ALP and a higher PTH level compared to young patients. This suggests better adaptability of young children to vitamin D deficiency compared with adolescents, and this may also mean that time is needed to develop clearer signs of vitamin D deficiency. The positive correlation between FEV_1_ and HtSDS suggests the importance of good lung function for normal linear growth in these children. In summary, CF patients with the CFTR I1234V mutation showed normal linear growth and BMI and a high prevalence of vitamin D deficiency, suggesting that pancreatic exocrine function plays a major role in maintaining normal growth in patients with CF. Measurements of circulating vitamin D and PTH concentrations appear to be helpful for the early detection of abnormal calcium homeostasis in these patients.
